# Complete mitochondrial genome of the roe deer *Capreolus pygargus tianschanicus* (Cervidae) from Korea

**DOI:** 10.1080/23802359.2017.1365645

**Published:** 2017-08-17

**Authors:** Hye Ri Kim, Mi Gyeong Jeon, Ji Hong Min, Hyun Ju Kim, Yung Chul Park

**Affiliations:** a Ecosystem Research Division, National Park Research Institute, Korea National Park Service, Wonju, Republic of Korea;; b Division of Forest Science, College of Forest & Environmental Sciences, Kangwon National University, Chuncheon, Republic of Korea;; c Microbial Safety Team, National Institute of Agricultural Sciences, Rural Development Administration, Wanju, Republic of Korea

**Keywords:** Mitogenome, roe deer, *Capreolus pygargus*, Cervidae

## Abstract

We determined and annotated the whole mtDNA genome of the roe deer *Capreolus pygargus tianschanicus* in Korea. The complete mitogenome is a circular molecule of 16,357 bp in length, including 13 protein-coding genes, 2 ribosomal RNA genes, 22 transfer RNA genes, and 2 non-coding regions (L-strand replication origin and control region). The mitogenome is AT-biased, with a nucleotide composition of 33.5% A, 30.0% T, 23.2% C, and 13.4% G. The phylogenetic analysis revealed the Korean roe deer *C. p. tianschanicus* is placed within the genus *Caprelous* clade, which has the water deer of the genus *Hydropotes* as sister clade.

The roe deer of the genus *Capreolus* (Cervidae) is widely distributed throughout the Palearctic and consists of two species, the smaller European roe deer *C. capreolus* and the larger Siberian roe deer *C. pygargus*. The Siberian roe deer is widespread species in the Palaearctic, several Eastern Europe, and continental Asia (Danilkin 1996; Matosiuk et al. 2014; Lee et al. 2015). The Siberian roe deer has been classified into three subspecies at regional level, *C. pygargus pygargus* (from Volga River to Lake Baikal and Northeastern Russia), *C. pygargus tianschanicus* (or *C. c. bedfordi*) (the Tian Shan Mountains, Mongolia, Russian Far East, and Korea) and *C. pygargus melanotis* (Eastern Tibet, and Gansu and Sichuan Province of China) (Lee et al. 2015).

We sequenced and annotated a mitogenome of *C. p. tianschanicus* in Korea. A fresh tissue for genomic DNA extraction was collected from the road-killed individual in agroecosystem of Odaesan National Park (N37 45 40.1, E128 36 44.3), South Korea. The voucher specimen (CECAPY-1) was deposited in the National Park Research Institute, Korea National Park Service. Genomic DNA extraction, PCR, and genome annotation were conducted according to the previous studies (Yoon et al. 2013; Jeon and Park 2015). A previously published mitogenome of *C. pygargus* (KJ681492) was used as a reference for gene annotation and primer design for PCR amplification of the Korean *C. pygargus* mitogenome. Phylogenetic tree was constructed using maximum-likelihood (ML) procedures implemented in MEGA6 (Tamura et al. 2013).

The complete mitogenome of the Korean roe deer (MF497305) contains total 16,357 bp length, which consists of a control region (one D-loop region) and a conserved set of 37 genes including 13 protein-coding genes (PCGs), 22 tRNA genes, and 2 ribosomal RNA genes (*12S rRNA* and *16S rRNA*). The mitogenome is AT-biased, with a nucleotide composition of 33.5% (5475 bp) A, 30.0% (4908 bp) T, 23.2% (3790 bp) C, and 13.4% (2184 bp) G. The 22 tRNA genes range from 60 bp (*tRNA^Ser(AGY)^*) to 75 bp (*tRNA^Leu(UUR)^*) in size. Lengths of the two rRNA genes and control region are 955 (*12S rRNA*), 1567 bp (*16S rRNA*), and 928 bp (control regions), respectively. The majority of 13 PCGs (nine of 13PCGs) use ATG as start codon, whereas *Nd2*, *Nd3*, and *Nd5* initiate with ATA and *Nd4L* starts with GTG, respectively. The incomplete stop codons (TA- or T–) are used for termination of *Nd1, Nd2,* and *Nd3* (T–) and *Nd4* and *Cox3* (TA-). TAG and AGA are used as stop codons in *Atp8* and *Cytb*, respectively, and the other six genes end with TAA. The replication origin *O_L_*is located between *tRNA^Asn^ and tRNA^Cys^* within the WANCY tRNA cluster as seen in most vertebrates (Kim and Park 2012; Kim et al. 2013; Yoon et al. 2013). In the phylogenetic tree based on mitogenome sequences (Figure 1), the Korean roe deer is well grouped with the roe deer from Russia and Poland and placed within the *Caprelous* clade, which has the water deer of the genus *Hydropotes* as sister clade.

**Figure 1.**
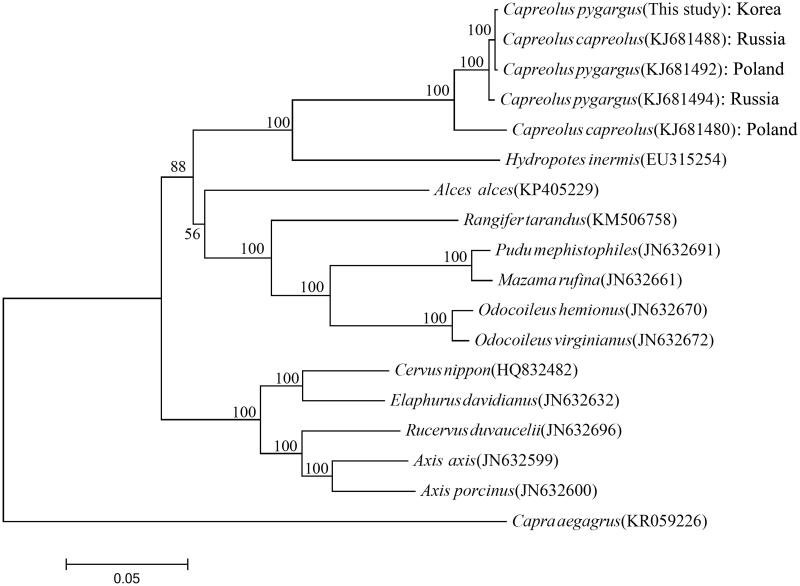
The phylogenetic position of the Korean roe deer *C. pygargus tianschanicus* inferred from maximum-likelihood analysis based on mitogenome sequences. The ML tree was generated using the GTR + G + I model, and the robustness of the tree was tested with 1000 bootstrap replicates. The numbers on the branches indicate bootstrap values.
